# Ageing in humans: separating intrinsic ageing from lifestyle effects

**DOI:** 10.1186/1471-2474-16-S1-S9

**Published:** 2015-12-01

**Authors:** Janet M Lord, Niharika A Duggal, Ross Pollock, Norman Lazarus, Stephen Harridge

**Affiliations:** 1 MRC-ARUK Centre for Musculoskeletal Ageing Research, University of Birmingham, Birmingham B15 2TT, UK; 2 Centre of Human and Aerospace Physiological Sciences, King's College London, London, UK

## 

We are an ageing society, with falling birth rates and increasing life expectancy. However healthy life span is not keeping pace and on average older adults can expect to be unwell for the last decade of life. Many factors influence both lifespan and healthspan but in humans one of the key factors is likely to be increasing physical inactivity. To separate out those elements of the ageing phenotype that are due to inactivity from those which are intrinsic to the ageing process, we recruited 125 adults aged 55-79 who had maintained a high level of physical activity through their adult lives. We compared these with age matched healthy older adults who were not involved in regular physical activity and healthy young subjects. The subjects were assessed for key features known to change with ageing including sarcopenia, reduced bone mineral density, adiposity, cardiovascular and lung function as well as markers of immune ageing. We have already reported the physiological data which revealed that many features of ageing including lean body mass, adiposity and muscle strength did not change with age in the physical active group, though other effects were seen such as a decline in lung capacity (FEV1) and maximal heart rate. Here we report that a comparison of immune phenotype in the exercising and non-exercising groups showed that thymic output was significantly reduced in the inactive group compared to both the young subjects (p<0.002) or the physically active older subjects (p<0.009). The numbers of naïve T cells was also maintained in the active elders, though the rise in the numbers of senescent T cells was not protected by an active lifestyle. We conclude that an active lifestyle through adulthood can prevent many of the physiological and immune features normally attributed to ageing.

